# Qualitative and Quantitative Metabolite Comparison of Korean Traditional Alcoholic Beverages: *Takju*, *Yakju*, and *Traditional-Soju*

**DOI:** 10.3390/foods13060956

**Published:** 2024-03-21

**Authors:** Hyun-Ji Tak, Na-Rae Lee, Sowon Yang, So-Young Kim, Choong-Hwan Lee

**Affiliations:** 1 Department of Bioscience and Biotechnology, Konkuk University, Seoul 05029, Republic of Korea; cathytak95@gmail.com (H.-J.T.); ysw6575@naver.com (S.Y.); 2 Research Institute for Bioactive-Metabolome Network, Konkuk University, Seoul 05029, Republic of Korea; michelle3690@gmail.com; 3 Department of Agrofood Resources, National Institute of Agricultural Sciences, Rural Development Administration, Wanju 55365, Republic of Korea; foodksy@korea.kr

**Keywords:** Korean traditional alcoholic beverages, non-targeted metabolite profiling, quantitative metabolite analysis, organic acids, amino acids, fatty acids

## Abstract

With increasing interest in Korean foods and beverages, Korean traditional alcoholic beverages need to be studied. To characterize Korean traditional alcoholic beverages, we analyzed the metabolites of *Takju*, *Yakju*, and *Traditional-Soju* using 48 commercial products. We performed non-targeted metabolite profiling using gas chromatography time-of-flight mass spectrometry (GC-TOF-MS) and identified 33 significantly discriminant metabolites, including nine organic acids, three amino acids, and seven fatty acids, in the three types of alcoholic beverage. Subsequently, we quantified the profiled metabolites in each product and compared their contents to identify alcoholic beverage type-specific metabolites. Thus, we figured out seven metabolites using receiver operating characteristic (ROC) curves. The results revealed that octadecanoic acid (limit of detection (LOD) to 168.72 mg/L), nonanoic acid (LOD to 112.54 mg/L), and octanoic acid (8.00 to 145.08 mg/L) in *Takju*; succinic acid (LOD to 1.90 mg/mL), heptanoic acid (LOD to 343.23 mg/L), and hexadecanoic acid (20.28 to 126.45 mg/L) in *Yakju*; and malonic acid (LOD to 19.13 mg/mL) in *Traditional-Soju*, with an area under the curve (AUC) > 0.7, are important metabolites that can distinguish the type of alcoholic beverage. Our results provide qualitative and quantitative metabolite information about Korean traditional alcoholic beverages that can be used by consumers and manufacturers.

## 1. Introduction

Alcoholic beverages have been consumed by humans for approximately 10,000 years. Over the years, numerous types of alcohol have been prepared using a variety of ingredients (e.g., wheat, rice, grape, apple, etc.) and manufacturing methods (e.g., brewing, distilling, aging) in different regions. Recently, the alcoholic beverage market has continued to grow and globalize owing to the development of transportation and cultural interests [1,2]. As part of the globalization of the alcoholic beverage market, Korean traditional alcoholic beverages are receiving interest because of the Korean Wave [3].

**Figure 1:**
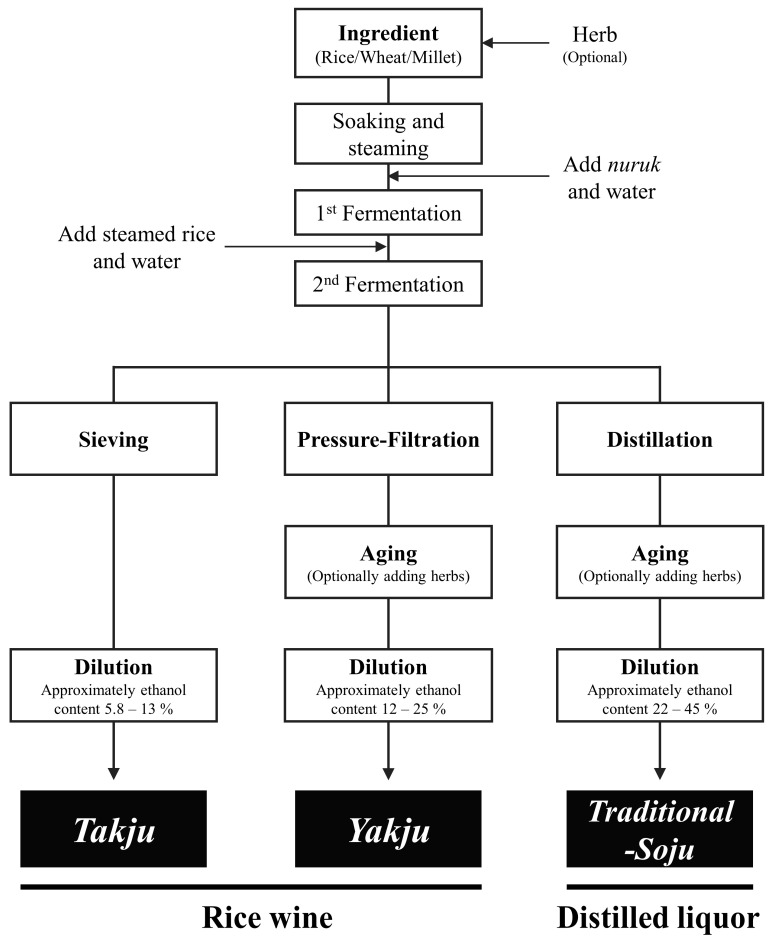
General manufacturing process of three Korean traditional alcoholic beverages, *Takju*, *Yakju*, and *Traditional-Soju*.

Korean traditional alcoholic beverages comprise *Takju*, *Yakju*, and *Traditional-Soju*, which are fermented using grains (e.g., rice, wheat, and millet) and *nuruk* as main ingredients [4]. *Nuruk* is a traditional culture starter made of grains and microorganisms such as *Aspergillus* spp., lactic acid bacteria (LAB), and yeasts (e.g., *Saccharomyces cerevisiae*) [5,6]. The types of Korean traditional alcoholic beverage are classified as *Takju*, *Yakju*, and *Traditional-Soju* depending on the manufacturing process (e.g., distillation and filtration) (Figure 1). *Takju*, also known as *makgeolli* (milky rice wine), is thick, unrefined, and opaque, with salty, umami, and yogurt-like tastes [7,8]. This is produced by filtering the fermented grain solution through a sieve [9]. *Yakju* is a clear rice wine with diverse flavors [10]. It is produced by pressure filtering the fermented grain solution. *Traditional-Soju* is almost transparent, with rich and spicy flavors, and has a higher alcohol content than *Takju* and *Yakju* [11]. It is produced by batch distilling liquid fermented grains such as rice, wheat, and millet. The three types of alcoholic beverage can be sorted into rice wine (*Takju*, *Yakju*) and distilled liquor (*Traditional-Soju*) depending on whether the fermented grain solution is distilled or not.

The flavors and tastes of beverages are affected by low-molecular weight compounds (e.g., organic acids, amino acids, and fatty acids). In particular, the contents of nutrients, flavors, and tastes are enhanced by fermentation [12,13]. For instance, a previous study reported that the levels of lysine and alanine, eliciting a sweet taste, and leucine and phenylalanine, eliciting a bitter taste, were dramatically increased during *doenjang* fermentation [14]. Ye et al. also determined the contents of organic acids, such as malic acid and lactic acid, that contribute to the sourness of fermented apple juice [15].

Metabolomics is a powerful approach used to understand the metabolic characteristics of organisms by analyzing metabolite changes [16]. This approach has been harnessed to analyze foods and beverages, such as juice and fermented foods (*doenjang*, cheese, wine, and beer) [17,18,19,20]. Regarding fermented foods, many studies have tried to understand the metabolite composition changes during fermentation. Seo et al. studied the metabolite changes in *makgeolli* with fermentation or aging time [21]. Furthermore, He et al. observed the metabolomic perturbation in liquor during distillation [22]. However, a comprehensive analysis of both non-targeted and targeted metabolites has not been performed to compare various fermented grain alcoholic beverages.

Therefore, in this study, we observed the metabolite differences in 48 Korean traditional alcoholic beverages using a metabolomics approach. First, using a non-targeted metabolomics approach by gas chromatography time-of-flight mass spectrometry (GC-TOF-MS) analysis, we profiled the metabolites in *Takju*, *Yakju*, and *Traditional-Soju*. We then quantified the organic acids, amino acids, and fatty acids that can contribute to the tastes and flavors of alcoholic beverages based on the profiled metabolites. Furthermore, alcoholic beverage type-specific metabolites were figured out using receiver operating characteristic (ROC) curves [23].

## 2. Materials and Methods

### 2.1. Chemicals and Reagents

High-performance liquid chromatography (HPLC)-grade water, acetonitrile, and methanol were purchased from Fisher Scientific (Pittsburgh, PA, USA). Potassium dihydrogen phosphate, sodium dihydrogen phosphate dihydrate, sodium hydroxide, trichloroacetic acid, and boric acid were obtained from Junsei Chemical Co., Ltd. (Tokyo, Japan). Formic acid, *N*-methyl-*N*-(trimethylsilyl) trifluoroacetamide (MSTFA), methoxyamine hydrochloride, pyridine, hydrochloric acid solution, potassium tetraborate tetrahydrate, o-phthaldialdehyde (OPA), 3-mercaptopropionic acid (3-MPA), 9-fluorenylmethoxycarbonyl chloride (FMOC-Cl), isooctane, and all standard compounds were purchased from Sigma-Aldrich (St. Louis, MO, USA).

### 2.2. Alcoholic Beverage Samples

Forty-eight alcoholic beverage products, classified into three groups (*Takju*, *Yakju*, and *Traditional-Soju*), were selected to evenly cover seven different provinces of South Korea. We purchased three bottles for each product from local markets, with 15 *Takju* products, 20 *Yakju* products, and 13 *Traditional-Soju* products. Detailed information (e.g., sample number, province of manufacture, alcohol content, and major ingredients) of all products is presented in Table S1. Fifty milliliters of sample were aliquoted from each bottle and stored at 4 °C until metabolite analysis.

### 2.3. Sample Extraction for Non-Targeted Metabolomic Analysis

To assess metabolomic differences in alcoholic beverages, we prepared a mixture for each type of alcoholic beverage by pooling 10 µL of each product. The pooled samples were dried using a speed vacuum concentrator (Biotron, Seoul, Republic of Korea) and re-dissolved in methanol containing an internal standard solution (2-chloro-l-phenylalanine, 1 mg/mL in water) to adjust the concentration to 5 mg/mL. The solution was sonicated for 5 min and filtered using 0.2 μm polytetrafluoroethylene (PTFE) syringe filters (Chromdisc, Daegu, Republic of Korea).

### 2.4. GC-TOF-MS Analysis for Non-Targeted Analysis

One hundred microliters of each re-dissolved solution was aliquoted into 1.5 mL Eppendorf tubes and completely dried using a speed vacuum concentrator. The dried extract was derivatized as follows in two steps: First, oximation was performed by adding 50 μL of methoxyamine hydrochloride in pyridine (20 mg/mL) to the dried extract, followed by incubation at 30 °C for 90 min. Next, silylation was performed by adding 50 μL of MSTFA to the oximated sample, followed by incubation at 37 °C for 30 min. The derivatized extracts were filtered using a 0.2 μm PTFE syringe filter prior to analysis. One microliter of derivatized sample was injected into the GC-TOF-MS with a split ratio of 15:1. The GC-TOF-MS analysis was performed using an Agilent 7890B GC system (Agilent Technologies, Palo Alto, CA, USA) with an Agilent 7693 autosampler (Agilent Technologies) and a Pegasus BT TOF-MS (LECO, St. Joseph, MI, USA). To separate the metabolites, an Rtx-5MS capillary column (30 m × 0.25 mm × 0.25 μm particle size, Restek Corp., Bellefonte, PA, USA) was used with helium as the carrier gas at a 1.0 mL/min flow rate. The GC column oven temperature was set to 75 °C for 2 min, increased to 300 °C at a rate of 15 °C/min, and then maintained at 300 °C for 3 min. The detector voltage was 2065 V, and masses were collected within the 50–600 *m*/*z* range. The injector and ion source temperatures were both set at 250 °C. All experiments were performed in triplicate.

### 2.5. Quantification of Organic Acids

An ultra-high-performance liquid chromatography-diode array detector (UHPLC-DAD) was used for organic acid quantification. Each alcoholic beverage sample was diluted 10 times using water, and then filtered through a 0.2 μm PTFE syringe filter. Subsequently, 5 μL of internal standard solution (levulinic acid, 1 mg/mL in water) was added to 100 μL of filtered sample before instrumental analysis [24,25]. The analyte separation was conducted using an ACQUITY UPLC BEH C18 column (150 mm × 2.1 mm × 1.7 μm particle size; Waters Corp., Milford, MA, USA) with an oven temperature of 30 °C. The injection volume was 2 μL. The mobile phase was 0.02 mol/L potassium dihydrogen phosphate (KH_2_PO_4_) mixed with 0.2% of acetonitrile (*v*/*v*), adjusted to pH 2.6 with 5% sulfuric acid solution. The flow rate was 0.1 mL/min. For organic acid detection, the diode array detector was set at 212 nm. The UHPLC system was equipped with a Vanquish binary pump F system (Thermo Fisher Scientific, Waltham, MA, USA) coupled with a Vanquish Split Sampler FT (Thermo Scientific) and DIONEX ULTIMATE 3000 RS DAD (Thermo Scientific). Each organic acid was quantified using standard curves of chromatographically pure compounds, including pyruvic acid, glucuronic acid, succinic acid, lactic acid, malonic acid, malic acid, tartaric acid, gluconic acid, and citric acid. Each standard curve was generated within the range mentioned in Table S3. The limit of detection (LOD) and quantification (LOQ) values of each metabolite were calculated using the standard deviation of the response (σ) and slope of the calibration curve (S) by using the formula: LOD  =  3.3 σ/S and LOQ  =  10 σ/S.

### 2.6. Quantification of Amino Acids

We used a high-performance liquid chromatography-diode array detector (HPLC-DAD) system to quantify the amino acids in the samples. Amino acid extraction and derivatization methods were based on previous studies with slight modifications [26,27,28]. To precipitate protein in the samples, 300 μL of each alcoholic beverage was mixed with 250 μL of 10% trichloroacetic acid and centrifuged at 13,000 rpm for 5 min. After that, 200 μL of supernatant was mixed with 250 μL of saturated potassium tetraborate to adjust to pH 9.5, followed by centrifugation at 13,000 rpm for 5 min. The resulting supernatant (200 μL) was filtered through a 0.2 μm PTFE syringe filter and derivatized using OPA/3-MPA and FMOC-Cl as follows: First, the same amounts of OPA solution (20 mg/mL, dissolved in 0.4 N boric acid) and 3-MPA solution (20 mg/mL, dissolved in 0.4 N boric acid) were mixed. Then, 4 μL of OPA/3-MPA mixed solution and 4 μL of internal standard solution (L-norvaline, 1 mg/mL in 0.1 mol/L HCl) were added to 4 μL of filtered supernatant. After 1 min, 20 μL of 0.4 N borate buffer and 4 μL of FMOC-Cl solution (2.5 mg/mL in acetonitrile) were added to the sample mixture. Subsequently, 108 μL of water was added to the mixture and the resultant mixture was filtered using a 0.2 μm PTFE syringe filter, and then the sample was used for HPLC-DAD analysis. Five microliters of the sample was injected. A Zorbax Eclipse-AAA column (4.6 × 150 mm, 5 μm with guard column) was used for separation at an oven temperature of 40 °C. Mobile phase A was 0.04 mol/L sodium dihydrogen phosphate (NaH_2_PO_4_), adjusted to pH 7.8 with 10 N sodium hydroxide (NaOH) solution. Acetonitrile-methanol-water (45:45:10, *v*/*v*/*v*) was used as mobile phase B. The separation was performed at a flow rate of 2 mL/min use a solvent gradient (vol.%) as follows: 0 min, 0% B; 2.9 min, 0% B; 19.1 min, 57% B; 19.6 min, 100% B; 23.3 min, 100% B; 24.2 min, 0% B; and 30 min, 0% B. The DAD was set at 338 nm. The HPLC system was equipped with a Chromaster 5110 pump (HITACHI, Tokyo, Japan) coupled with a Chromaster 5280 auto sampler (HITACHI) and a Chromaster 5430 diode array detector (HITACHI). Similar to organic acids, each amino acid was quantified using standard curves of chromatographically pure compounds, including alanine, phenylalanine, and leucine. The standard curves, LOD, and LOQ of the quantified metabolites were derived as described above.

### 2.7. Quantification of Fatty Acids

Quantitative analysis of the fatty acids was performed using GC-TOF-MS. The alcoholic beverage samples (100 μL for *Traditional-Soju*, 50 μL for *Takju* and *Yakju*) were dried using a speed vacuum concentrator and dissolved in isooctane, owing to the polarity of fatty acids, containing internal standard solution (behenic acid, 1 mg/mL in isooctane) to give 10 mg/mL solutions [29]. The solution was sonicated for 5 min and filtered using a 0.2 μm PTFE syringe filter [30]. The derivatization and analytical methods used were consistent with those in the previously mentioned non-targeted analysis using GC-TOF-MS. Each fatty acid was quantified using standard curves of chromatographically pure compounds, including *cis*-9-octadecenoic acid, octadecanoic acid, decanoic acid, nonanoic acid, octanoic acid, heptanoic acid, and hexadecenoic acid. Similar to other metabolites, the standard curves, LOD, and LOQ of the quantified fatty acids were calculated using the same methods.

### 2.8. Data Processing and Multivariate Statistical Analysis

The raw GC-TOF-MS data were converted to NetCDF (*.cdf) files using LECO ChromaTOF software (version 4.44). Peak detection, retention time correction, and alignment were performed using the MetAlign software package version 1.0.0.1 (http://www.metalign.nl, accessed on 23 March 2023). Subsequently, metabolites were tentatively identified by comparing the mass retention times, fragment patterns, and mass analysis data with standard compounds under identical conditions or using available databases, such as the National Institutes of Standards and Technology Library (NIST; version 2.0, 2011; FairCom, Gaithersburg, MD, USA), PubChem (https://pubchem.ncbi.nlm.nih.gov/), and the Human Metabolome Database (HMDB; https://hmdb.ca/). Multivariate statistical analyses, including principal component analysis (PCA) and partial least squares discriminant analysis (PLS-DA), were performed using SIMCA-P+ software (version 15.0.2; Umetrics, Umea, Sweden). Discriminant metabolites were selected based on their variable importance in the projection (VIP) value > 1.0, based on a PLS-DA score plot, and significant differences (*p*-value < 0.05) were tested by one-way ANOVA using STATISCA (version 7.0, StaSoft Inc., Tulsa, OK, USA). ROC curves and one-way ANOVA with Duncan’s multiple-range test were generated using PASW Statistics 18 (SPSS Inc., Chicago, IL, USA) to evaluate significant differences in the quantification results.

## 3. Results

### 3.1. Non-Targeted Metabolite Profiling of Takju, Yakju, and Traditional-Soju

**Figure 2:**
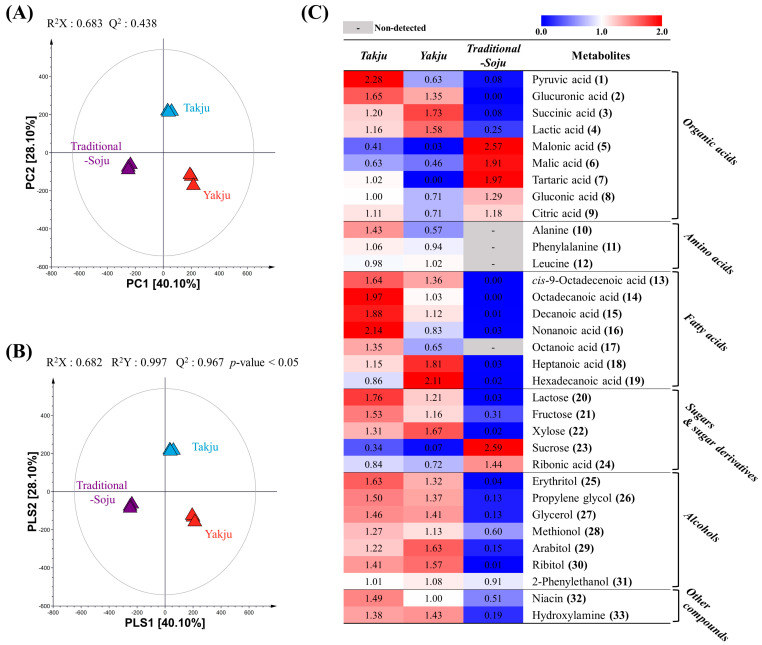
Principal component analysis (PCA) score plot (**A**) and partial least squares discriminant analysis (PLS-DA) score plot (**B**) of *Takju* (▲), *Yakju* (▲), and *Traditional-Soju* (▲) based on the GC-TOF-MS analysis results. Heat map analysis of three types of alcoholic beverage based on GC-TOF-MS data (**C**). The heatmap represents the relative abundance of significantly discriminant metabolites (VIP > 1.0, *p*-value < 0.05) based on the PLS-DA model. The numbers in parentheses represent the number of identified metabolites. The score plots and heatmap data are normalized to the raw material weight.

We performed metabolite profiling using GC-TOF-MS to identify significantly different metabolites in the three types of Korean traditional alcoholic beverage. Subsequently, multivariate statistical analyses using the GC-TOF-MS analysis results were conducted to evaluate the differences in the three types of alcoholic beverage. All non-targeted metabolomic analysis data were normalized by the raw material volume because the different kinds of alcoholic beverages showed different extraction yields. The resultant PCA and PLS-DA score plots are presented in Figure 2A,B. The PCA revealed the obvious separation of *Traditional-Soju* from *Takju* and *Yakju* by PC1 (40.10%) (Figure 2A). Moreover, *Takju* was distinguished from *Yakju* and *Traditional-Soju* by PC2 (28.10%). A similar pattern was observed in the PLS-DA score plot (Figure 2B).

We investigated significantly discriminant metabolites among the three different types of alcoholic beverage based on the VIP value (VIP > 1.0) and *p*-value (<0.05) using the PLS-DA model and one-way ANOVA, respectively. Consequently, 33 metabolites were tentatively identified, including nine organic acids, three amino acids, seven fatty acids, five sugars and their derivatives, seven alcohols, and two others (Table S2). The relative concentrations of the 33 metabolites are illustrated in Figure 2C. The abundance of organic acids differed depending on the type of beverage. For example, pyruvic acid (1) and glucuronic acid (2) were relatively abundant in *Takju*. Succinic acid (3) and lactic acid (4) showed the highest amounts in *Yakju* compared to other beverages, while malonic acid (5), malic acid (6), tartaric acid (7) and gluconic acid (8) showed the highest amounts in *Traditional-Soju*. Interestingly, amino acids, fatty acids, and alcohols were abundant in rice wine, whereas their levels were low in *Traditional-Soju*, even with amino acids (10–12) and octanoic acid (17) not detected. Among the fatty acids observed, *cis*-9-octadecenoic acid (13), octadecanoic acid (14), decanoic acid (15), nonanoic acid (16), and octanoic acid (17) were more abundant in *Takju* than in *Yakju*, whereas heptanoic acid (18) and hexadecanoic acid (19) had relatively higher amounts in *Yakju*. Among the sugars, lactose (20), fructose (21), and xylose (22) were abundant in rice wine, whereas levels of sucrose (23) and ribonic acid (24) were comparatively higher in the distilled liquor, *Traditional-Soju*.

### 3.2. Metabolic Pathways of Three Korean Traditional Alcoholic Beverages

**Figure 3:**
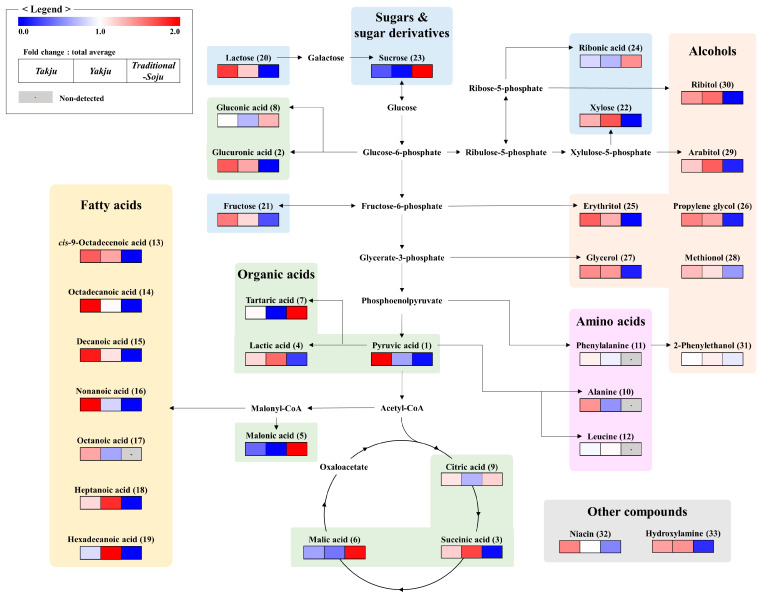
Schematic diagram of the metabolic pathways and relative levels of metabolites in *Takju*, *Yakju*, and *Traditional-Soju*. The pathways were modified based on the Kyoto Encyclopedia of Genes and Genomes (KEGG) database (http://www.genome.jp/kegg/). Discriminant metabolites among the alcoholic beverages were selected by VIP value (>1.0) and *p*-value (<0.05) based on the PLS-DA model. The colored squares (blue-to-red) represent the relative abundance of each metabolite. Each column represents the fold-change values normalized to an average value in all datasets.

To comprehend the fermentative characteristics of the three different types of alcoholic beverage, we reconstructed their metabolic pathways using the relative contents of significantly different metabolites (Figure 3). In rice wine, sugars and sugar alcohols (21, 22, 25, 27, 29, 30), other alcohols (26, 28), and organic acids (2) derived from the glycolysis pathway showed relatively higher contents than in distilled liquor. Pyruvic acid (1) and lactic acid (4), synthesized from pyruvic acid, were comparatively abundant in rice wine. Moreover, three amino acids (10–12) derived from phosphoenolpyruvate or pyruvic acid were also comparatively abundant in rice wine. Unlike other pyruvic acid derived metabolites, tartaric acid (7) abundance was higher in distilled liquor.

The relative contents of fatty acids (13–19) synthesized from acetyl-CoA were relatively higher in rice wine. On the other hand, malonic acid (5), also derived from acetyl-CoA, showed a higher content in distilled liquor. Interestingly, each organic acid (3, 6, and 9) in the tricarboxylic acid (TCA) cycle showed different patterns.

### 3.3. Quantitative Analysis of Organic Acids, Amino Acids, and Fatty Acids

**Figure 4:**
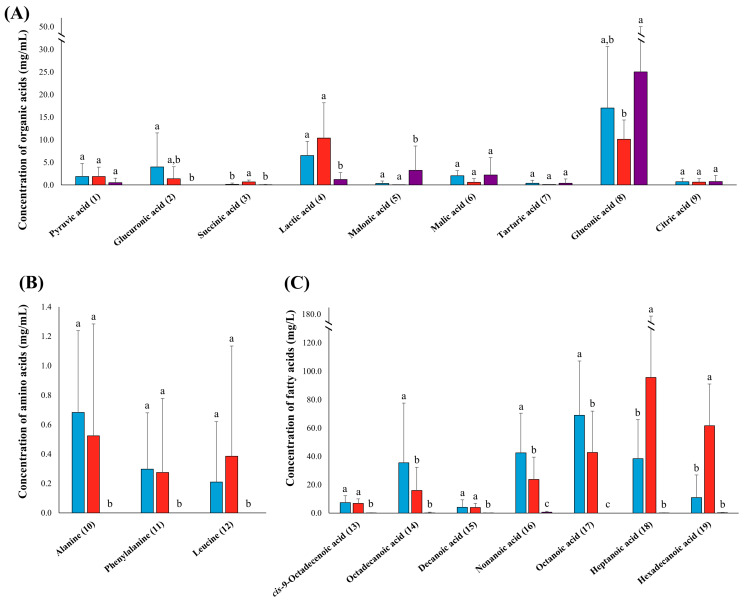
Quantitative analysis data of organic acids (**A**), amino acids (**B**), and fatty acids (**C**) in *Takju* (■), *Yakju* (■), and *Traditional-Soju* (■). Different letters above the bars indicate significant differences according to Duncan’s multiple-range test (*p*-value < 0.05).

The significantly different organic acids, amino acids, and fatty acids in the three types of alcoholic beverage, which were revealed using non-targeted metabolite analysis, were quantified based on the standard curve of each metabolite (Tables S3–S6) by harnessing suitable analytical methods for each class of metabolite. Furthermore, the average amounts of each metabolite in the three types of alcoholic beverage were presented as bar graphs, with error bars representing the deviation of the metabolite concentrations within each product in the three types of alcoholic beverage (Figure 4A–C).

Intriguingly, the gluconic acid (8) content was highest among the measured metabolites in all three types of alcoholic beverage (Figure 4A). The quantified amount of gluconic acid was within the range of LOD to 46.75 mg/mL in *Takju*, 2.61 to 17.82 mg/mL in *Yakju*, and 0.36 to 80.16 mg/mL in *Traditional-Soju* (Tables S4–S6). Lactic acid (4) showed a relatively higher content in *Yakju*; ranging from 0.25 to 27.35 mg/mL in *Yakju*, 1.18 to 12.22 mg/mL in *Takju*, and LOD to 5.17 mg/mL in *Traditional-Soju*. The next highest-content metabolites were glucuronic acid (2) in *Takju* and malonic acid (5) in *Traditional-Soju*, with ranges of LOD to 23.40 mg/mL and LOD to 19.13 mg/mL, respectively. Succinic acid (3) had a content range of LOD to 1.90 mg/mL in *Yakju*, which was higher than that in the other alcoholic beverage types. The other organic acids exhibited similar levels in all three types of alcoholic beverage. Similar to non-targeted metabolite analysis, alanine (10), phenylalanine (11), and leucine (12) were only quantified in rice wine, although amounts of these amino acids were lower than the LOQ in distilled liquor (Figure 4B). The quantitative analysis results showed no statistically significant differences in the levels of each amino acid in *Takju* and *Yakju*. Surprisingly, the contents of fatty acids were very low compared to organic acids and amino acids (Figure 4C). The fatty acid contents were also comparatively abundant in rice wine, whereas negligible amounts of fatty acids existed in distilled liquor, which was consistent with the results of the non-targeted analysis. Notably, among the fatty acids, octadecanoic acid (14), nonanoic acid (16), and octanoic acid (17) were relatively abundant in *Takju*, ranging from LOD to 168.72 mg/L, LOD to 112.54 mg/L, and 8.00 to 145.08 mg/L, respectively, compared to other types of alcoholic beverage. On the other hand, heptanoic acid (18) and hexadecanoic acid (19) were the most abundant fatty acids in *Yakju*, compared to the other alcoholic beverage types, within the range of LOD to 343.23 mg/L and 20.28 to 126.45 mg/L, respectively.

**Figure 5:**
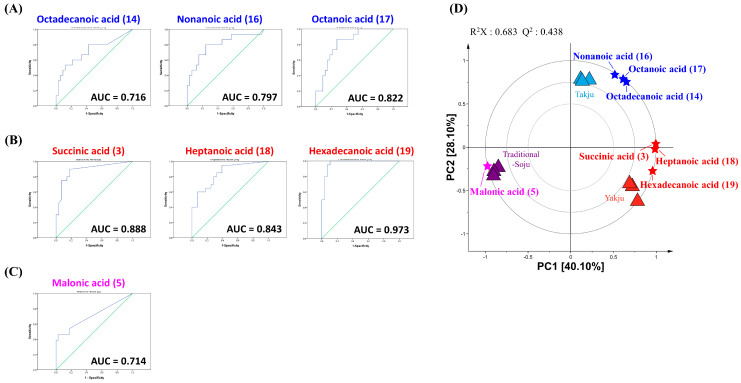
Receiver operating characteristic (ROC) curve of metabolites distinguishing one type of alcoholic beverage from the others (**A**–**C**). (**A**) *Takju*, (**B**) *Yakju*, and (**C**) *Traditional-Soju*. The area under the curve (AUC) values of each metabolite are shown inside the ROC curve. (**D**) PCA biplot of *Takju* (▲), *Yakju* (▲), and *Traditional-Soju* (▲) derived from the non-targeted analysis using GC-TOF-MS. The PCA biplot represents the relativity of selected metabolites with three different types of alcoholic beverage. Selected metabolites in *Takju* (★), *Yakju* (★), and *Traditional-Soju* (★).

To figure out unique metabolites for distinguishing each type of alcoholic beverage, we performed one-way ANOVA with a post hoc test and ROC curve analysis using the quantification results of each product. As a result, we selected unique metabolites that showed statistically higher amounts in each type of alcoholic beverage than in others and exhibited good discriminatory power with an area under the curve (AUC) > 0.7, resulting in seven metabolites as follows: octadecanoic acid (14), nonanoic acid (16), and octanoic acid (17) in *Takju* (Figure 5A); succinic acid (3), heptanoic acid (18), and hexadecanoic acid (19) in *Yakju* (Figure 5B); and malonic acid (5) in *Traditional-Soju* (Figure 5C). Subsequently, to validate the uniqueness of the selected seven metabolites in *Takju*, *Yakju*, and *Traditional-Soju*, we conducted PCA biplot analysis using the non-targeted metabolite analysis results (Figure 5D). The plot showed clustering patterns between the selected metabolites and type of alcoholic beverage, implying the representativeness of each metabolite with each type of alcoholic beverage.

## 4. Discussion

Korean traditional alcohols have recently been in the spotlight worldwide. To understand the characteristics of taste and flavor in alcoholic drinks, it is essential to figure out the distinguished metabolites in each type of alcoholic beverage. Although a previous study analyzed the chemical properties (e.g., pH, acidity) and organic acid contents of *Takju*, *Yakju*, and *Soju*, no studies have compared their metabolites using both qualitative and quantitative analyses [31]. In this study, we comprehensively analyzed three types of Korean traditional alcoholic beverage and subsequently quantified significantly discriminant metabolites among the three types of alcoholic beverage, which were selected based on the non-targeted analysis. As a result, we could discern the characteristics of each type of alcoholic beverage.

The multivariate statistical analyses using non-targeted metabolite analysis showed clear separation of rice wine from distilled liquor by PC1 and PLS1 in PCA and PLS-DA, respectively, implying that the distillation process highly affects the metabolite contents in alcoholic beverages (Figure 2A,B). Distillation can separate and concentrate metabolites from fermented grain solution according to the characteristics of the metabolites [32]. Furthermore, *Takju* and *Yakju* were distinguished by PC2 and PLS2 in PCA and PLS-DA, respectively, which are produced by different filtration methods (e.g., sieve and pressure filtration). Based on the multivariate statistical analysis, we found significantly discriminant metabolites among the three alcoholic beverages (Figure 2C and Figure 3).

In our results, distilled liquor mainly contained some organic acids and sugars, while most amino acids, fatty acids, sugar alcohols, and other organic acids and sugars were abundant in rice wine due to the high boiling points of most metabolites [33]. For instance, heptanoic acid and succinic acid were not quantified in most *Traditional-Soju* products (Table S6) since they have high boiling points, as observed consistently in Jeong et al.’s results [31]. Moreover, numerous metabolites that exist in fermented grain solution are esterified by the distillation process, indeed affecting them as flavor compounds [34,35,36]. Furthermore, additives (e.g., herbs and fruits) can change the metabolite composition of alcoholic beverages. The distilled liquors without additives, S05, S09, S10, S12, and S13, showed gluconic acid as a major organic acid. On the other hand, in S02 and S06, licorice and mulberry were added during the manufacturing process, respectively, and they contained other organic acids (e.g., malonic acid, malic acid, tartaric acid, and citric acid) along with gluconic acid (Table S1) [37,38]. The important metabolites differing based on the filtration method were the fatty acids, particularly heptanoic acid and hexadecanoic acid, which were abundant in most *Yakju* products (Figure 4C and Table S5). Consistent results were observed in the non-targeted analysis (Figure 2C).

We assumed that ethanol production in alcoholic beverages might be mainly performed by *Saccharomyces cerevisiae*, one of the representative yeasts in *nuruk* [39]. *Aspergillus* spp., one of the major fungi in *nuruk*, might synthesize gluconic acid (8), imparting a refreshing taste, as a major product that we observed as the most abundant metabolite in most Korean traditional alcoholic beverages (Figure 4A) [5,40,41]. LAB, which is also inoculated via *nuruk*, could be a main contributor to lactic acid (4) production, which might originate from pyruvic acid (1) and malic acid (6) (Figure 4A) [42,43]. The richness of lactic acid can contribute to the sour taste of alcohol [25]. Other metabolites might be produced and/or consumed through the interaction of microorganisms present in *nuruk* that contribute to the flavors and tastes of alcoholic beverages [44].

The seven unique metabolites, selected via ROC curve analysis, can decipher the flavors and tastes of each type of alcoholic beverage (Figure 5A–C). The combination of unique compounds can create the characteristic taste and flavor of each alcoholic beverage. The yogurt-like taste of *Takju* might be generated by the combination of the three unique metabolites, octadecanoic acid (14), nonanoic acid (16), and octanoic acid (17), eliciting a tallow-like taste, creamy milk flavor, and sweet-like fruity odor, respectively [25,45]. *Yakju* showed one organic acid and two fatty acids as unique metabolites: succinic acid (3), heptanoic acid (18), and hexadecanoic acid (19) (Figure 5B) [46]. Succinic acid, heptanoic acid, and hexadecanoic acid have a salty-bitter taste, fatty odor, and mildly sweet taste, respectively, giving the harmonious flavor of *Yakju* [47,48,49]. *Traditional-Soju* has only one unique metabolite, malonic acid (5), which increases the sourness of *Traditional-Soju* (Figure 5C) [50,51]. We assumed that *Traditional-Soju* contained relatively higher amount of malonic acid than rice wine since it might be condensed by distillation and/or originated from additives. Through ROC curve analysis, we were able to figure out unique metabolites that can help us understand the characteristics of each type of alcoholic beverage (Figure 5).

Even though we investigated significantly different metabolites among the three types of alcoholic beverage, volatile compounds in the alcoholic beverages were not analyzed in this study. Further study is needed to analyze volatile compounds in alcoholic beverages to comprehensively understand odor. In addition, it is necessary to study the metabolomic changes in alcoholic beverages which produced under designed conditions to understand the effects of ingredients and manufacturing processes.

## 5. Conclusions

Even though Korean foods and beverages have received interest globally, the characteristics of Korean traditional alcoholic beverages have not been fully explored so far. Therefore, in this study, we characterized the metabolite differences in 48 commercial products of *Takju*, *Yakju*, and *Traditional-Soju*, using non-targeted and targeted analysis. Non-targeted metabolomic analysis revealed that the relative contents of most amino acids, fatty acids, sugar alcohols, and some organic acids and sugars were abundant in *Takju* and *Yakju*, while those of other organic acids and sugars were comparatively higher in *Traditional-Soju*. Furthermore, the compositions of fatty acids were different between *Takju* and *Yakju*. The metabolite differences were highly correlated with the distillation process and filtration method. Subsequently, we conducted quantitative analysis using each product and tried to find unique metabolites that can contribute to the tastes and flavors of the alcoholic beverages. As a result, we figured out seven unique metabolites that can decipher the flavor and taste of each type of the three alcoholic beverages. This study may help to understand the characteristics of Korean traditional alcoholic beverages and can be used to improve the quality of products. In addition, it can be used as a cornerstone of Korean traditional foods and beverages toward globalization.

## Data Availability

The original contributions presented in the study are included in the article/Supplementary Materials, further inquiries can be directed to the corresponding author.

## Supplementary Materials

The following supporting information can be downloaded at: https://www.mdpi.com/article/10.3390/foods13060956/s1, Table S1: Sample information for *Takju*, *Yakju*, and *Traditional-Soju*; Table S2: Discriminative metabolites in *Takju*, *Yakju*, and *Traditional-Soju* derived from the PLS-DA model based on the GC-TOF-MS analysis; Table S3. Standard curve equations of organic acids, amino acids, and fatty acids; Table S4: The concentrations of organic acids, amino acids, and fatty acids in *Takju*; Table S5: The concentrations of organic acids, amino acids, and fatty acids in *Yakju*; Table S6: The concentrations of organic acids, amino acids, and fatty acids in *Traditional-Soju*.
